# The relationship between complete blood cell count-derived inflammatory biomarkers and benign prostatic hyperplasia in middle-aged and elderly individuals in the United States: Evidence from NHANES 2001–2008

**DOI:** 10.1371/journal.pone.0306860

**Published:** 2024-07-09

**Authors:** Chengdong Shi, Hongliang Cao, Guoqiang Zeng, Lei Yang, Yuantao Wang

**Affiliations:** Department of Urology II, The First Hospital of Jilin University, Changchun, China; Yarmouk University, JORDAN

## Abstract

**Background:**

Benign prostatic hyperplasia (BPH) is a common health disorder of the male genitourinary system with a high prevalence, especially among middle-aged and older adults, which seriously affects men’s quality of life. Inflammatory markers derived from complete blood cell count (CBC) have previously been considered a prognostic indicator for various diseases, but little is known about their relationship with BPH. This study evaluated the relationship between complete blood cell count (CBC)-derived inflammatory biomarkers and BPH.

**Methods:**

Data for this cross-sectional study were gathered from the National Health and Nutrition Examination Survey (NHANES) between 2001 and 2008. Using multiple logistic regressions, the study examined the association between benign prostatic hyperplasia(BPH) and Inflammatory biomarkers derived from blood cell counts such as neutrophil-to-lymphocyte ratio (NLR), platelet-to-lymphocyte ratio (PLR), monocyte-to-lymphocyte ratio (MLR), Systemic Inflammatory Response Index (SIRI) and Systemic Immunoinflammatory Index (SII).

**Results:**

3,919 participants were included, with a median age of 61.00 (52.00–71.00) years old. Among them, 609 participants had benign prostatic hyperplasia, with a prevalence of 15.54%. Upon accounting for confounding factors, the study revealed a positive correlation between the plurality of BPH PLR and SII. However, MLR, NLR, and SIRI did not significantly correlate with the prevalence of BPH (p>0.05). In contrast to the lowest quartile, higher quartiles of PLR (OR = 1.93[1.38–2.69]) and SII (OR = 1.71[1.22–2.40]) were linked to an elevated risk of BPH. Interaction tests showed that age, body mass index, hypertension, diabetes, smoking, and drinking had no significant effect on this positive correlation (p for interaction>0.05). In addition, we found a roughly linear association between SII, PLR, and BPH using smoothed curve fitting.

**Conclusions:**

According to our research, high levels of PLR and SII are positively linked with an increased risk of BPH in middle-aged and elderly individuals in the United States. The results compensate for previous studies that still need to be validated with larger prospective cohorts.

## 1 Introduction

The prostate gland is a crucial part of the male reproductive system, responsible for urinary control and reproductive functions. Benign Prostatic Hyperplasia (BPH) commonly affects middle-aged and older men. As men age, the likelihood of developing BPH increases, with 50–75% of men over 50 years experiencing prostatic hyperplasia [1]. BPH can cause prostatic bladder obstruction (BOO) and lower urinary tract symptoms (LUTS), which can significantly impact a patient’s quality of life [2, 3]. BPH etiology remains uncertain, but inflammation and immune responses may be involved, with various inflammatory factors playing an important role. Inflammatory infiltrates (mostly T-lymphocyte infiltrates, with an increase in the expression of B-lymphocytes and macrophages) have been found in many prostate enlargement patients who have undergone surgery [4, 5]. Inflammatory markers are promising as biomarkers for evaluating the severity of the condition and identifying potential treatment targets for affected patients [6].

Recently, Different CBC-derived inflammatory markers like neutrophil-to-lymphocyte ratio (NLR), platelet-to-lymphocyte ratio (PLR), monocyte-to-lymphocyte ratio (MLR), Systemic Inflammatory Response Index (SIRI), and Systemic Immunoinflammatory Index (SII) have been employed as prognostic indicators across various illnesses [7, 8]. These biomarkers are a set of comprehensive prognostic parameters that combine peripheral platelets, lymphocytes, and neutrophils. They respond more comprehensively to the immune-inflammatory status of the host than a single inflammatory indicator [9, 10]. A meta-analysis by Linghao Meng et al. showed that high levels of SII were associated with poor survival and progression-free survival in patients with prostate cancer. Therefore, it can be used as an important prognostic indicator for prostate cancer patients [11].

Moreover, SIRI was an independent predictor of survival outcomes in bladder cancer and was more potent than other inflammatory markers [12]. In addition, NLR and PLR were negatively correlated with spontaneous ureteral stone passage (SSP), which can be used along with other indices for clinical decision-making in ureteral stone treatment [13]. On the other hand, high levels of NLR and PLR were associated with poor overall survival in metastatic renal cell carcinoma, and NLR and PLR instead of neutrophil count and platelet count improved the predictive accuracy of the International Metastatic Renal Cell Carcinoma Database Consortium model (IMDC) [14]. There is a relative lack of research on the use of inflammatory biomarkers for diagnosing prostate enlargement. However, A recent single-center retrospective study evaluated the diagnostic efficiency of inflammatory biomarkers for BPH by subject workup traits and an artificial neural network. It concluded that PLR, NLR, and SII can be used to diagnose BPH [15]. Another study conducted by the TCLSIH Cohort found that higher levels of NLR might be associated with an increased risk of developing BPH in the adult male population in China, making it an essential target for BPH prevention and intervention [16]. Lastly, a study found that platelets may be a protective factor for patients with diabetes mellitus and BPH. They were negatively correlated with prostate volume and positively correlated with MLR [17].

It is currently believed that BPH may be associated with chronic inflammation. Still, the exact timing and cause of chronic inflammation are unknown, and the immune and inflammatory mechanisms at play might diverge from those seen in other medical conditions [18, 19]. Therefore, the relationship between inflammatory biomarkers derived from complete blood count (CBC) and benign prostatic hyperplasia (BPH) has yet to be thoroughly examined in patients. This study evaluates the connection between CBC-derived inflammatory biomarkers and BPH by analyzing a nationally representative sample from the 2001–2008 National Health and Nutrition Examination Survey (NHANES).

## 2. Materials and methods

### 2.1 Study population

**Fig 1:**
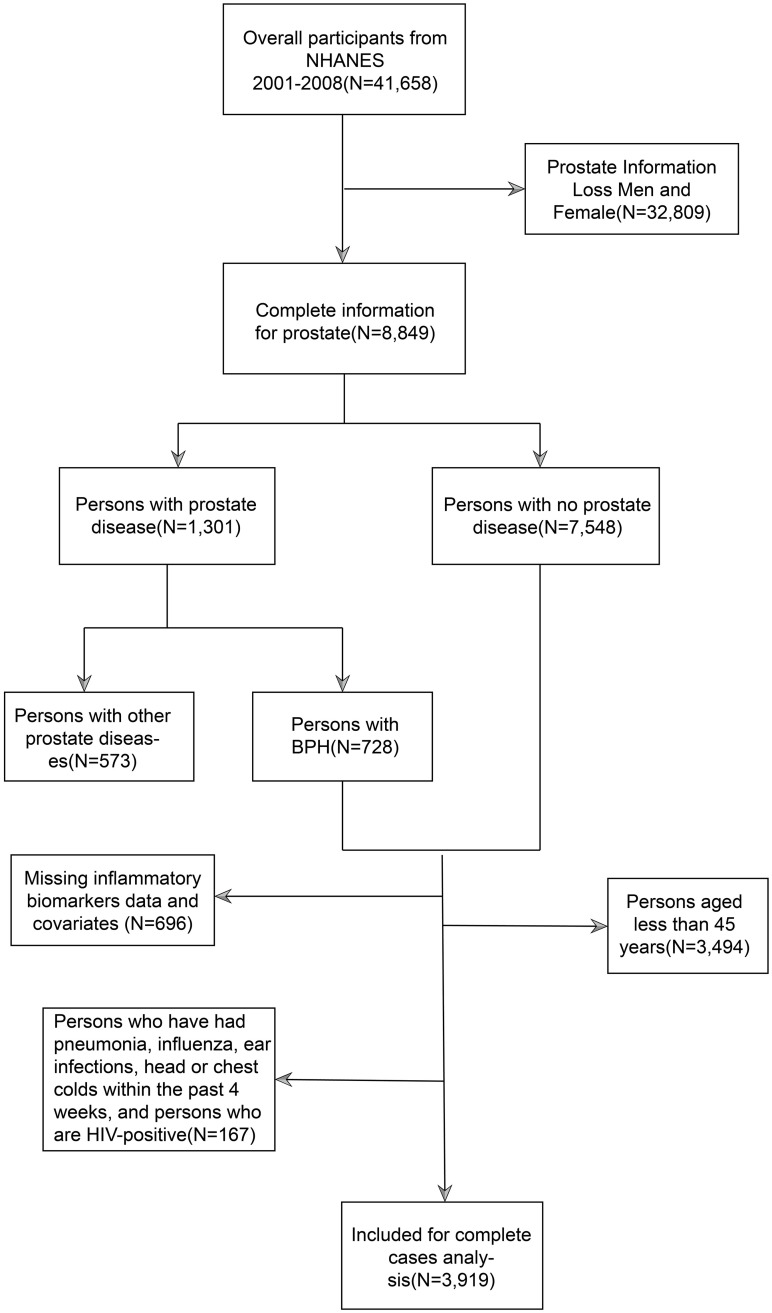
Flowchart of the participant selection from NHANES 2001–2008.

The current study utilized a cross-sectional design based on the NHANES dataset. The data within the NHANES database is managed by the National Center for Health Statistics (NCHS), part of the Centers for Disease Control and Prevention (CDC) in the United States [20]. The objective is to collect information concerning the health and nutrition of the American population, encompassing demographic, socio-economic, dietary, and health-related aspects [21]. The physical examination section includes physiological measurements and laboratory tests. The National Center for Health Statistics (NCHS) Institutional Review Board approved the research protocol, and all participants provided informed consent. From 2001 to 2008, a total of 41,658 individuals were recruited. Exclusion criteria comprised: (A) females and those with missing data for prostate assessment, (B) incomplete data for inflammatory markers (neutrophil, lymphocyte, and platelet counts) and covariates, (C) persons who are less than 45 years of age, (D) Persons who have had pneumonia, influenza, ear infections, head or chest colds within the past 4 weeks, and persons who are HIV-positive Ultimately, 3,919 participants were included in the comprehensive case analysis (Fig 1).

### 2.2 Assessment of CBC-derived inflammatory biomarkers

SIRI, SII, NLR, PLR, and MLR consist of peripheral lymphocytes (L), neutrophils (N), monocytes (M), and platelets (P) and are defined as SIRI = N x M/L (103/μL), SII = P x N/L (103/μL), NLR = N/L (103/μL), PLR = P/L (103/μL), and MLR = M/L (103/μL) [22].

### 2.3 Assessment of BPH

When male participants answered "No" to the question "Have prostate disease?" they were considered free of benign prostatic hyperplasia. If participants answered "Yes" to the question "Have prostate disease?" and responded "Yes" to both "Ever told had enlarged prostate?" and "Was it a benign enlargement?" they were classified as having benign prostatic hyperplasia. Participants falling into the following categories were excluded: other prostate diseases, non-benign prostate enlargement, unknown responses, refusal to answer questions, or missing values among males [23].

### 2.4 Covariates definition

Information regarding baseline data of participants was collected through a questionnaire survey and laboratory tests, including age (in years), race/ethnicity categories including Mexican American, other Hispanic, non-Hispanic White, non-Hispanic Black, or other racial backgrounds [24]. Education levels were categorized as below high school, high school graduate, or higher. Body mass index (BMI) was stratified into three groups: <25.0, 25.0–30.0, and ≥30 kg/m2 [25]. Income was evaluated using the poverty income ratio (PIR), calculated by dividing family income by the specific poverty threshold for that family size and classified as ≤1.0, 1.0–3.0, and >3.0, following guidelines from the US Department of Health and Human Services [26]. Alcohol consumption status was categorized as non-drinkers, low to moderate drinkers (≤3 drinks/day), or heavy drinkers (>3 drinks/day). Information on the prevalence of hypertension and diabetes was retrieved through self-reported questionnaires [27]. This method quantified c-reactive protein (CRP) by latex-enhanced nephelometry.

### 2.5 Statistical analysis

In describing the study group, continuous variables following a normal distribution were shown as mean ± standard error (SE), while those without a normal distribution were indicated by median (P25, P75). Categorical variables were displayed as percentages. PLR, NLR, MLR, SII, and SIRI were categorized into quartiles (Q1 to Q4). Variations between participants grouped by quartiles or the existence of BPH were evaluated using weighted t-tests (for continuous variables) or weighted chi-square tests (for categorical variables). To examine the correlation between PLR, NLR, MLR, SII, SIRI, and the presence of BPH, multivariable weighted logistic regression was conducted to determine the adjusted odds ratios (OR) and 95% confidence intervals (CI) for CBC-derived inflammatory biomarkers associated with the presence of BPH. The crude model had no covariates, model 1 adjusted for covariates age, race, and model 2 further adjusted for age, race, education level (below high school, high school or above), poverty income ratio (PIR) (≤1.0, 1.0–3.0, >3.0), alcohol consumption status (non-drinkers, low to moderate drinkers, heavy drinkers), smoking status (non-smokers, smokers), BMI (<25.0, 25.0–30.0, ≥ 30.0), CRP, diabetes, hypertension(Diabetes and hypertension were recorded based on self-report (indicating yes or no)). Smooth curve fitting was performed to explore the linear or non-linear associations between PLR, SII, SIRI, and BPH. Subgroup and interaction analyses were conducted based on age, PIR, BMI, hypertension, diabetes, alcohol consumption, and smoking. Statistical analyses were performed using R Studio (version 4.3.2) and Empowering Stats (version 2.0). A p-value < 0.05 (two-tailed) was considered statistically significant for differences.

## 3. Results

### 3.1 Characteristics of study participants

This study encompassed 41,658 participants from 2001 to 2008. Exclusions were made for female participants and those lacking prostate data (N = 32,809), followed by the exclusion via questionnaire responses of males reporting other prostate diseases, non-benign prostate enlargement, unknown reactions, or refusal to answer questions (N = 573). Subsequently, participants lacking data on inflammatory markers and covariates (N = 696) and those younger than 45 years (N = 3,494), as well as those with pneumonia, influenza, ear infections, head or chest colds in the past 4 weeks, and HIV-positive individuals (N = 167) were excluded, resulting in a final inclusion of 3,919 participants. Table 1 illustrates the baseline features of adults with inflammatory biomarkers within the NHANES dataset from 2001 to 2008. The median age of participants was 61.00 (52.00–71.00) years old, predominantly non-Hispanic Whites (58.25%). The median, upper quartile (P25), and lower quartile (P75) values for NLR, PLR, MLR, SIRI, and SII were 2.10 (1.57–2.81), 108.33 (53.58–148.18), 0.29 (0.23–0.38), 1.16 (0.80–1.69), and 407.24 (156.94–623.97), respectively. A total of 609 participants (15.54%) were diagnosed with BPH. In contrast to participants without BPH, individuals with BPH tended to be older non-Hispanic Whites with higher education levels and income, were more inclined to smoke, have moderate alcohol intake, be overweight, and suffer from hypertension (P<0.05). BPH patients also displayed notably higher white blood cell (WBC) and Lymphocyte counts (P<0.05). Substantial variances in all CBC-derived indices were observed between participants with and without BPH.

**Table 1:** Baseline variables of adults with CBC-derived inflammatory biomarkers in NHANES 2001–2008.

Variables	Total	BPH		*P* Value
		Yes	No	
Number	39,19	609	3,310	
Age, years	61.00 (52.00–71.00)	70.00 (62.00–78.00)	60.00 (51.00–69.00)	<0.001
Race, (%)				<0.001
Mexican American	16.23	10.02	17.37	
Other Hispanic	4.85	3.78	5.05	
Non-Hispanic White	58.25	72.90	55.56	
Non-Hispanic Black	18.07	12.15	19.15	
Other Race—Including Multi-Racial	2.60	1.15	2.87	
Education (%)				<0.001
Below high school	30.08	22.99	31.39	
High school	23.81	21.02	24.32	
Above high school	46.11	55.99	44.29	
Family PIR, (%)				<0.001
≤1.0	13.65	8.87	14.53	
1.0–3.0	40.70	41.70	40.52	
>3.0	45.65	49.43	44.95	
Smoking status, (%)				0.105
Smoker	65.27	68.14	64.74	
Non-smoker	34.73	31.86	35.26	
Alcohol drinking history (%)				<0.001
Nondrinker	35.20	54.69	31.87	
Low-to-moderate drinker	42.47	35.12	43.73	
Heavy drinker	22.33	10.19	24.40	
Diabetes, (%)				0.963
Yes	18.65	18.72	18.64	
No	81.35	81.28	81.36	
Hypertension, (%)				<0.001
Yes	44.63	53.04	43.08	
No	55.37	46.96	56.92	
Body mass index, (%)				0.908
<25.0 kg/m2	24.37	23.97	24.44	
25.0–30.0 kg/m2	43.20	44.01	43.05	
≥ 30.0 kg/m2	32.43	32.02	32.51	
CRP,mg/dl	0.20 (0.09–0.42)	0.19 (0.09–0.40)	0.20 (0.09–0.43)	0.268
CBC count, 10^3^/μL				
White blood cell	6.90 (5.60–8.10)	6.70 (5.60–7.90)	6.90 (5.62–8.20)	0.012
Lymphocyte	1.90 (1.50–2.40)	1.70 (1.40–2.20)	1.90 (1.50–2.40)	<0.001
Monocyte	0.60 (0.50–0.70)	0.60 (0.50–0.70)	0.60 (0.40–0.70)	0.771
Neutrophils	4.00 (3.20–5.00)	4.00 (3.20–4.90)	4.00 (3.10–5.00)	0.925
Platelet	214.00 (134.00–263.00)	212.50 (155.00–257.00)	214.00 (112.50–265.00)	0.714
CBC-derived indicators				
MLR	0.29 (0.23–0.38)	0.32 (0.25–0.40)	0.29 (0.23–0.37)	<0.001
PLR	108.33 (53.58–148.18)	116.67 (72.00–161.18)	106.52 (45.91–145.88)	<0.001
NLR	2.10 (1.57–2.81)	2.29 (1.69–3.10)	2.07 (1.56–2.77)	<0.001
SII,10^3^/μL	407.24 (156.94–623.97)	437.06 (230.97–666.67)	402.48 (135.20–608.96)	<0.001
SIRI,10^3^/μL	1.16 (0.80–1.69)	1.27 (0.87–1.82)	1.14 (0.79–1.66)	<0.001

Normally distributed continuous variables are described as means ± SEs, and continuous variables without a normal distribution are presented as medians [P25, P75]. Categorical variables are presented as numbers (percentages). Percentages reflect the survey-weighted. Abbreviations: PIR, poverty income ratio; NLR, neutrophil-to-lymphocyte ratio; PLR, platelet-to-lymphocyte ratio; MLR, monocyte-to-lymphocyte ratio; SIRI, systemic inflammatory response index; SII, systemic immune-inflammation index; BPH, benign prostatic hyperplasia; CBC, complete blood cell, CRP, C-reactive protein.

### 3.2 Relationships between CBC-derived biomarkers and the prevalence of BPH

Four groups were created from all CBC-derived indices, and each group’s connection with the incidence of BPH was evaluated (Table 2). The incidence of BPH and the CBC-derived indices (PLR and SII) showed a positive association in the crude model. This relationship remained statistically significant after adjusting for age and race. In Model 2, higher levels of PLR and SII were significantly associated with an increased prevalence of BPH. Following adjustment for all included confounders, the highest quartile versus lowest quartile odds ratios (OR) and 95% confidence intervals (CI) were as follows: PLR (OR = 1.93 [1.38, 2.69], P_trend_<0.0001) and SII (OR = 1.71 [1.22, 2.40], P_trend_ = 0.0023). Additionally, we analyzed the relationship between CBC parameters and the prevalence of BPH (S1 Table). In the crude model, only white blood cells and lymphocytes negatively correlated with the bulk of BPH. However, after adjusting for all confounding factors, we observed a significant association between white blood cells, lymphocytes, monocytes, and platelets and a higher prevalence of BPH.

**Table 2:** OR (95% CI) of the prevalence of BPH according to quartiles of complete blood cell (CBC)-derived inflammatory biomarkers among adults in NHANES 2001–2008.

	Quartiles of CBC-derived inflammatory biomarkers levels				*P*_*trend*_
	Quartile 1	Quartile 2	Quartile 3	Quartile 4	
NLR					
Range	<1.57	1.57–2.10	2.10–2.81	>2.81	
Crude	1.0[Reference]	1.14 (0.88, 1.48)	1.41 (1.09, 1.81)	1.71 (1.34, 2.20)	<0.0001
Model 1	1.0[Reference]	0.95 (0.72, 1.26)	1.04 (0.79, 1.37)	1.00 (0.76, 1.31)	0.8979
Model 2	1.0[Reference]	1.04 (0.72, 1.49)	1.22 (0.86, 1.74)	1.15 (0.81, 1.65)	0.3892
MLR					
Range	<0.23	0.22–0.29	0.27–0.37	>0.37	
Crude	1.0[Reference]	1.20 (0.92, 1.57)	1.53 (1.18, 1.98)	1.91 (1.48, 2.45)	<0.0001
Model 1	1.0[Reference]	0.99 (0.75, 1.32)	1.02 (0.77, 1.35)	0.94 (0.71, 1.24)	0.6200
Model 2	1.0[Reference]	0.92 (0.64, 1.32)	0.93 (0.65, 1.34)	0.88 (0.61, 1.26)	0.5255
PLR					
Range	<53.64	53.64–108.33	108.33–148.18	>148.18	
Crude	1.0[Reference]	1.08 (0.84, 1.40)	1.17 (0.91, 1.51)	1.58 (1.24, 2.02)	0.0003
Model 1	1.0[Reference]	1.21 (0.92, 1.58)	1.23 (0.95, 1.61)	1.52 (1.18, 1.96)	0.0015
Model 2	1.0[Reference]	1.09 (0.75, 1.58)	1.56 (1.10, 2.19)	1.93 (1.38, 2.69)	<0.0001
SII					
Range	<157.08	157.08–407.24	407.24–624.00	>624.00	
Crude	1.0[Reference]	1.14 (0.88, 1.46)	1.12 (0.87, 1.45)	1.49 (1.16, 1.89)	0.0016
Model 1	1.0[Reference]	1.33 (1.02, 1.74)	1.17 (0.90, 1.53)	1.35 (1.04, 1.74)	0.0461
Model 2	1.0[Reference]	1.33 (0.93, 1.90)	1.31 (0.93, 1.84)	1.71 (1.22, 2.40)	0.0023
SIRI					
Range	<0.80	0.80–1.16	1.16–1.69	>1.69	
Crude	1.0[Reference]	1.17 (0.90, 1.51)	1.32 (1.03, 1.71)	1.54 (1.20, 1.97)	0.0005
Model 1	1.0[Reference]	0.92 (0.70, 1.21)	0.86 (0.65, 1.13)	0.77 (0.58, 1.02)	0.0566
Model 2	1.0[Reference]	0.85 (0.60, 1.22)	0.76 (0.53, 1.08)	0.78 (0.54, 1.12)	0.2299

Data are presented as OR (95% CI) unless indicated otherwise; Model 1 was adjusted as age(continuous) and race (Mexican American, Other Hispanic, NonHispanic White, Non-Hispanic Black, or Other). Model 2 was adjusted as model 1 plus education level (below high school, high school, or above high school), family poverty income ratio (≤1.0,1.0–3.0, or >3.0), drinking status (non-drinker, low-to-moderate drinker, or heavy drinker), CRP, smoking status (never smoker, smoker, BMI (<25.0, 25.0–30.0, or ≥ 30.0), self-reported diabetes (yes or no), and self-reported hypertension (yes or no). Abbreviations: OR, Odds Ratio; CI, Confidence Interval; CRP, C-reactive protein; BMI, Body mass index.

### 3.3 Subgroup analysis

**Fig 2:**
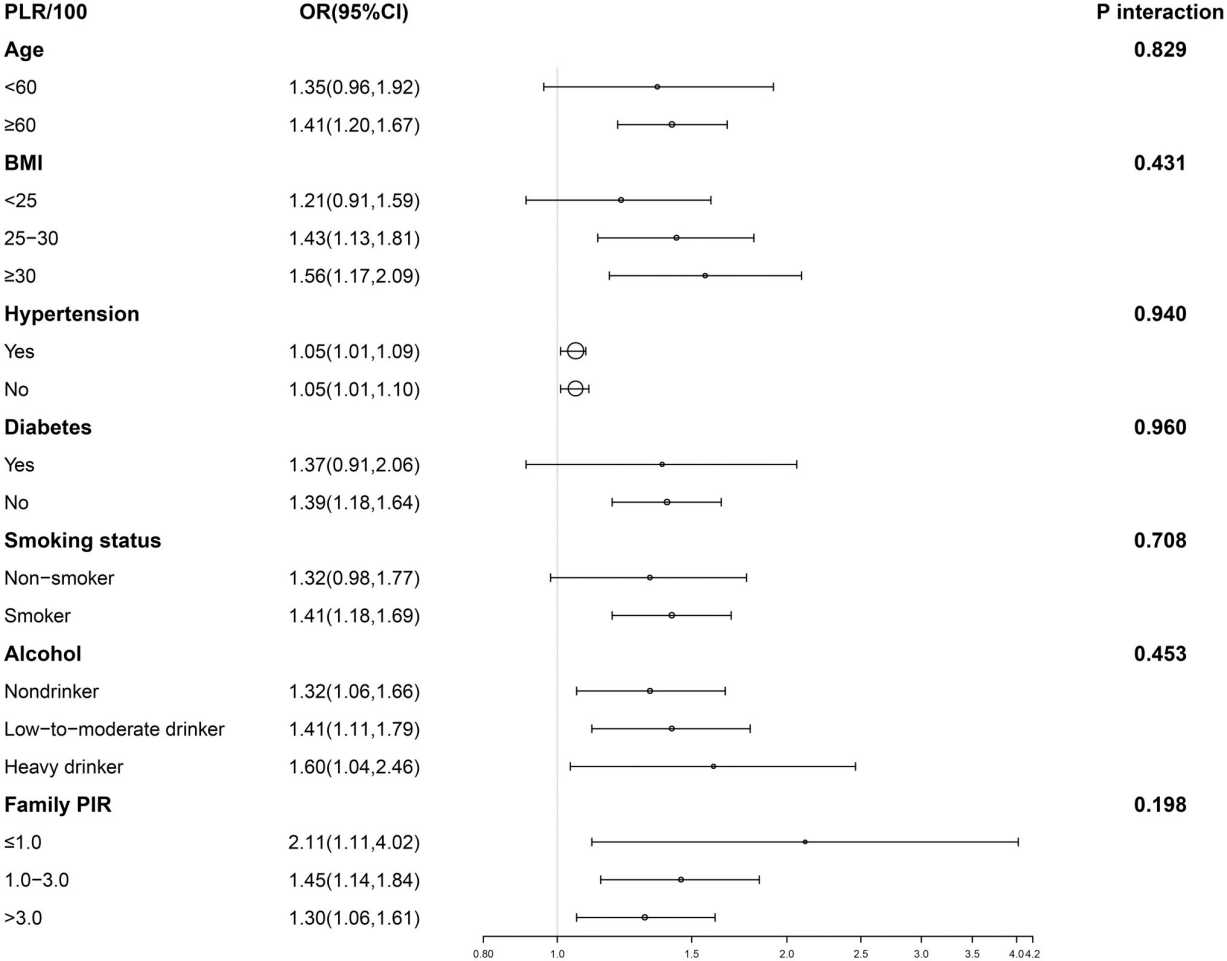
Subgroup analysis for the association between PLR/100 and BPH. Abbreviations PLR: Platelet-to-lymphocyte Ratio; BPH: Benign Prostatic Hyperplasia.

**Fig 3:**
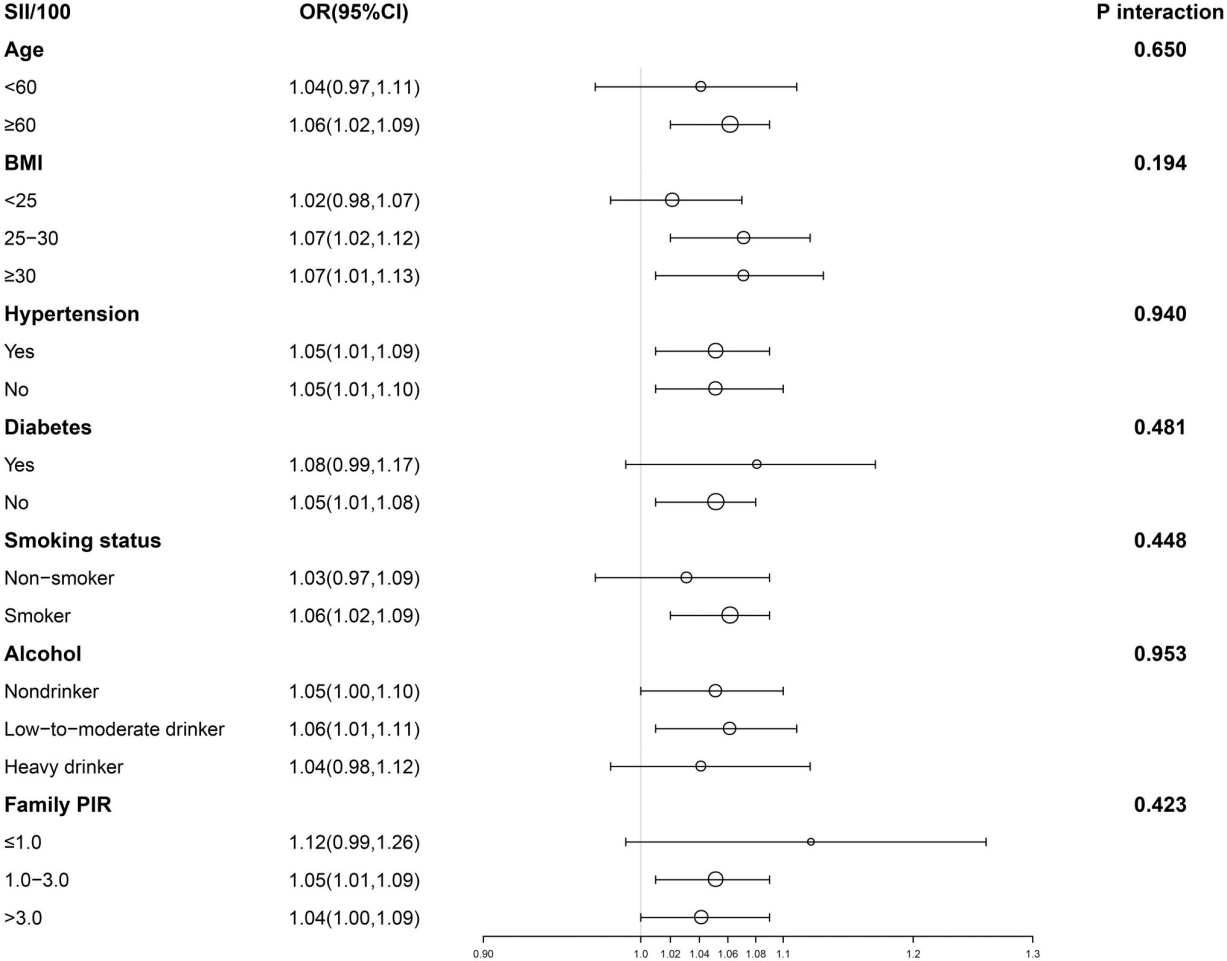
Subgroup analysis for the association between SII/100 and BPH. Abbreviations SII: Systemic Immunoinflammatory Index; BPH: Benign Prostatic Hyperplasia.

**Fig 4:**
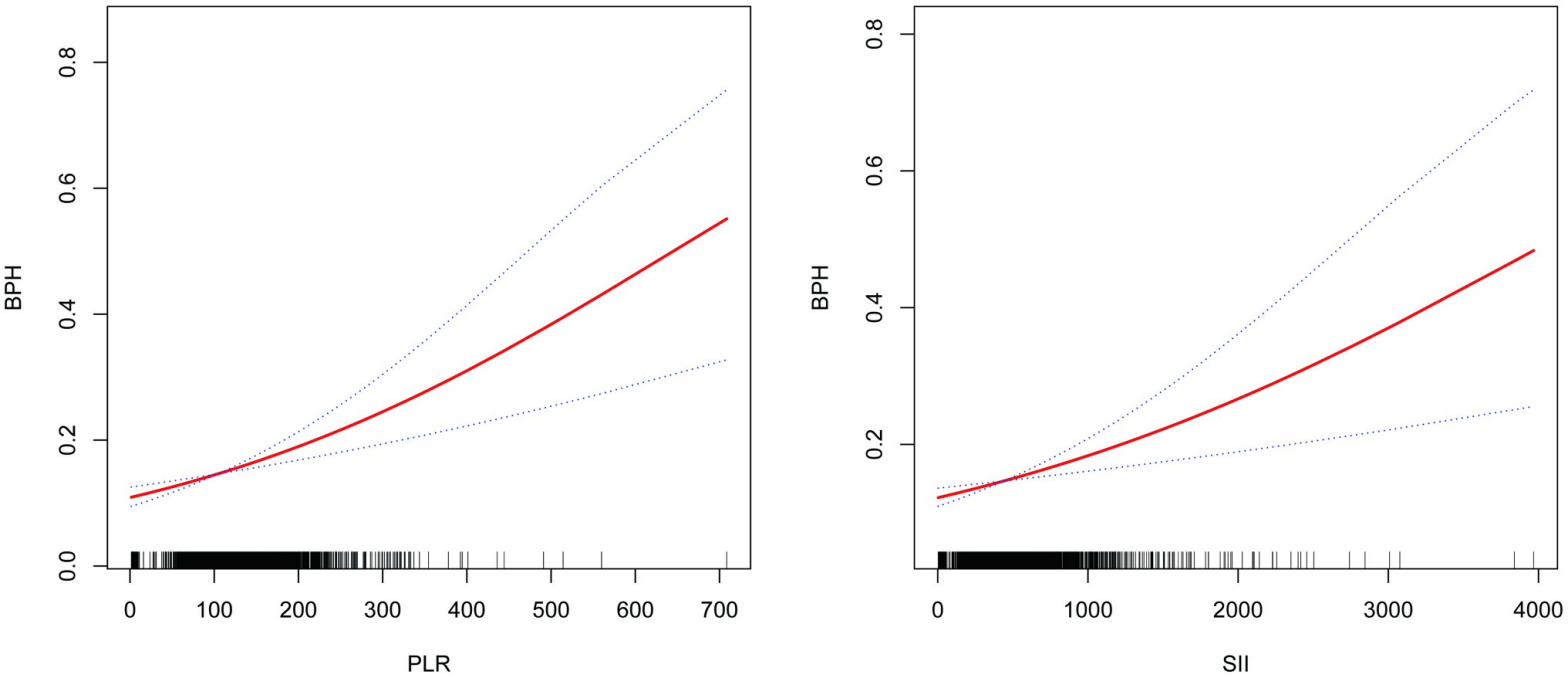
Association of PLR and SII with BPH. The solid red line represents the smooth curve fit between variables. The Blue dotted line represents the 95% confidence interval from the fit. Abbreviations PLR: Platelet-to-lymphocyte Ratio; BPH: Benign Prostatic Hyperplasia; SII: Systemic Immunoinflammatory Index.

Figs 2 and 3 display the subgroup analysis of the PLR, SII, and BPH association. Due to the subtle effect size, we magnified the values of SII and PLR by 100 and compared SII/100 and PLR/100 with the relationship to BPH. SII/100 exhibited a significant correlation with no diabetes, presence or absence of hypertension, PIR between 1 and 3, BMI ≥25, age at least 60, Low-to-moderate drinker, and smoking (p<0.05). PIR, diabetes, alcohol consumption, smoking, hypertension, age, and BMI did not significantly influence this positive association (P for interaction >0.05). PLR/100 displayed a significant correlation with no diabetes, PIR, BMI ≥25, presence or absence of alcohol consumption, age at least 60, with or without high blood pressure, and smoking (p<0.05). PIR, diabetes, alcohol consumption, smoking, hypertension, age, and BMI did not significantly impact this positive association (P for interaction >0.05). Subsequently, smooth curve fitting was used to describe whether there was a linear relationship between PLR, SII, and BPH. The findings indicated a roughly linear positive correlation between PLR, SII, and BPH (Fig 4).

## 4. Discussion

We conducted a cross-sectional study of 39,19 adult men to investigate the association of CBC-derived inflammatory biomarkers with the prevalence of BPH in the US population from 2001 to 2008. Our findings indicated that PLR and SII levels were significantly associated with most BPH, whereas MLR, NLR, and SIRI did not correlate. After adjusting for relevant confounders, we found that PRL and SII were positively related to the occurrence of BPH. The association among PLR, SII, and BPH was then characterized employing smoothing curve fitting; PLR and SII had a roughly linear correlation with the occurrence of BPH. According to these results, CBC-derived biomarkers of inflammation may be clinical tools for assessing the risk of developing BPH in adult men. In conclusion, our study emphasizes that consideration of the inflammatory state reflected by CBC-derived markers may be significantly associated with the development of BPH.

Benign prostatic hyperplasia (BPH) is a highly prevalent clinical condition, yet its pathogenesis remains unclear. The role of inflammation in BPH was first proposed in 1937, but the theory of embryonic reawakening was more widely accepted [6, 28]. Presently, the role of inflammation in BPH has reemerged as a focal point of research. Some researchers suggest that BPH represents an immune-mediated inflammatory condition, as nearly all surgical specimens from prostatic hyperplasia patients exhibit infiltration of inflammatory cells, primarily lymphocytes, macrophages, and mast cells [4, 19, 29]. The molecular mechanisms involving chronic inflammatory signals in its pathogenesis remain unclear. The prostate is an organ with an active immune system [30]. Theoretically, stimuli from exogenous or endogenous antigens may induce the prostate to initiate an inflammatory response, prompting the generation of inflammatory and growth factors by inflammatory cells, activating immune responses in T cells and macrophages, and releasing more inflammatory factors. Simultaneously, increased oxygen consumption due to cell proliferation creates a relatively hypoxic environment. The infiltration of inflammatory cells and the relatively hypoxic environment exacerbate tissue damage, promoting prostatic tissue’s healing, regeneration, and stromal expansion [31, 32].

CBC-derived indices are commonly used markers reflecting the immune and inflammatory status of the body, gaining attention in clinical settings due to their simplicity and cost-effectiveness. These indices have recently been utilized as prognostic factors for various conditions. In a study exploring the preoperative systemic immune-inflammation index (SII) in patients undergoing radical nephroureterectomy (RNU) for upper urinary tract urothelial carcinoma (UTUC), alterations in preoperative SII were significantly associated with higher pathological stages and poorer survival outcomes in UTUC RNU-treated patients [33]. Ran Zuo et al. found that the pretreatment systemic immune-inflammation response index (SIRI) in patients receiving first-line treatment for advanced lung adenocarcinoma (LUAD) served as an independent prognostic factor for progression-free survival (PFS) in patients. Dynamic monitoring of inflammatory index changes aids in predicting treatment efficacy [7]. Additionally, a meta-analysis study suggested that NLR and PLR are practical and convenient peripheral inflammatory markers for assessing glioblastoma prognosis [34].

However, there’s limited research on CBC-derived indices in benign prostatic hyperplasia (BPH). A propensity score-matched analysis indicated a significant association between high NLR and the presence of BPH, suggesting a potential influence of inflammation on the development of BPH [35]. Studies by Kutan Ozer et al. demonstrated a positive correlation between NLR and severe symptoms and BPH progression, hinting at the potential utility of anti-inflammatory therapy in BPH management [36]. According to our findings, elevated PLR and SII were positively associated with a higher risk of prostate enlargement, whereas SIRI exhibited a negative correlation with an increased risk of BPH. We speculate this association involves a cascade reaction, where neutrophils, lymphocytes, monocytes, and platelets play pivotal roles in initiating and regulating innate and adaptive immune responses. The cytokines secreted by these cells may have intricate connections with the occurrence and Progression of BPH. Multiple studies suggest elevated expression of pro-inflammatory cytokines in BPH tissue, yet the precise mechanisms of these inflammatory cytokines in BPH remain unclear [37, 38]. Research by Penn et al. indicated that stromal cells in benign prostatic hyperplasia could activate specific CD4+ T cells to produce IFNγ and IL-17, inducing the secretion of key growth factors IL-6 and IL-8 by epithelial cells and prostate stromal cells [39].

Additionally, pro-inflammatory cytokines and chemokines may recruit lymphocytes and monocytes as antigen-presenting cells, leading to an upregulation of specific cytokines and creating a vicious cycle that amplifies inflammation. Some studies have found overexpression of IL-17 in most BPH tissues, primarily produced by activated T cells and endothelial cells. IL-17 can stimulate BPH stromal cells to make up to 9 times more IL-6 and up to 26 times more IL-8 [40]. In conclusion, cytokines play a crucial role in immune-inflammatory-induced damage to prostatic tissues, but a deeper understanding of the mechanisms involved requires further investigation.

Our study has provided insights into the association between blood-derived inflammatory markers and BPH. PLR and SII serve as comprehensive inflammatory markers, better reflecting the interplay between innate and adaptive immune responses. Furthermore, these indices are clinically economical and readily accessible, offering convenience for clinicians in assessing BPH. They may also provide valuable insights into the immunoinflammatory mechanisms underlying BPH occurrence.

Compared to prior studies, our research possesses several strengths. Firstly, it relies on NHANES data, offering a more representative sample with a larger size, enabling the discovery of significant associations between independent and dependent variables. Secondly, we adjusted for confounding covariates to ensure the robustness of the current findings. Lastly, CBC-derived indices composed of multiple CBC parameters may offer more comprehensive information than a single index. However, our study has inherent limitations. It is a cross-sectional study, limiting our ability to establish definitive causal relationships.

Moreover, specific NHANES data outcomes rely on self-reported patient information, possibly introducing recall bias. Additionally, while we adjusted for various confounding factors, unidentified confounders and potential confounding factors not included in the NHANES database might still influence the analysis. Finally, our application of CBC parameters to compute CBC-derived indices in participants may introduce bias.

## 5. Conclusions

This cross-sectional study suggested high levels of PLR and SII were positively linked with an increased risk of BPH in middle-aged and elderly individuals in the United States and that this association was roughly linear. The results compensate for previous studies that still need to be validated with larger prospective cohorts.

## Supporting information

S1 Table OR (95% CI) of the prevalence of BPH according to CBC parameters among adults in NHANES 2001–2008. (DOCX)
