# Supplementary material for: The relationship between complete blood cell count-derived inflammatory biomarkers and benign prostatic hyperplasia in middle-aged and elderly individuals in the United States: Evidence from NHANES 2001–2008
Source: PLoS One. 2024 Jul 9;19(7):e0306860. doi: 10.1371/journal.pone.0306860 (PMC11233019; doi:10.1371/journal.pone.0306860)
Supplement: S1 Table — (DOCX) [file pone.0306860.s001.docx]

**S1 Table. OR (95% CI) of the prevalence of BPH according to CBC parameters among adults in NHANES 2001-2008**

|  | Crude | Model 1 | Model 2 |
| --- | --- | --- | --- |
|  | OR (95% CI)  p-Value | OR (95% CI)  p-Value | OR (95% CI)  p-Value |
| White blood cell | 0.96 (0.92, 1.00)  0.0484 | 0.94 (0.90, 0.99) 0.0133 | 0.93 (0.86, 0.99) 0.0274 |
| Lymphocyte | 0.86 (0.76, 0.97) 0.0130 | 0.98 (0.92, 1.04) 0.5132 | 0.79 (0.66, 0.94) 0.0098 |
| Neutrophils | 0.97 (0.92, 1.03) 0.3267 | 0.92 (0.86, 0.98) 0.0072 | 0.97 (0.89, 1.06) 0.4463 |
| Monocyte | 0.97 (0.63, 1.47) 0.8724 | 0.44 (0.27, 0.72) 0.0011 | 0.24(0.12, 0.48) <0.0001 |
| Platelet | 1.00 (1.00, 1.00) 0.1821 | 1.00 (1.00, 1.00) 0.0061 | 1.00 (1.00, 1.00) 0.0002 |

Data are presented as OR (95% CI) unless indicated otherwise; Model 1 was adjusted as age(continuous), race (Mexican American, Other Hispanic, NonHispanic White, Non-Hispanic Black or Other). Model 2 was adjusted as model 1 plus education level (below high school, high school,or above high school), family poverty income ratio (≤1.0,1.1–3.0, or >3.0), CRP, drinking status (nondrinker, low-to-moderate drinker, or heavy drinker), smoking status (never smoker, smoker, BMI (<25.0, 25.0-30.0, or ≥ 30.0),self-reported diabetes (yes or no), and self-reported hypertension (yes or no).
